# Independent functions of DNMT1 and USP7 at replication foci

**DOI:** 10.1186/s13072-018-0179-z

**Published:** 2018-02-27

**Authors:** Olya Yarychkivska, Omid Tavana, Wei Gu, Timothy H. Bestor

**Affiliations:** 1 Department of Genetics and Development, College of Physicians and Surgeons, Columbia University, 701 W. 168th St, New York, NY 10032 USA; 2 Department of Pathology and Cell Biology, Institute for Cancer Genetics, College of Physicians and Surgeons, Columbia University, New York, NY 10032 USA

**Keywords:** DNMT1, USP7, Glycine-lysine repeats, Protein stability, Replication foci

## Abstract

**Background:**

It has been reported that USP7 (ubiquitin-specific protease 7) prevents ubiquitylation and degradation of DNA methyltransferase 1 (DNMT1) by direct binding of USP7 to the glycine-lysine (GK) repeats that join the N-terminal regulatory domain of DNMT1 to the C-terminal methyltransferase domain. The USP7-DNMT1 interaction was reported to be mediated by acetylation of lysine residues within the (GK) repeats.

**Results:**

We found that DNMT1 is present at normal levels in mouse and human cells that contain undetectable levels of USP7. Substitution of the (GK) repeats by (GQ) repeats prevents lysine acetylation but does not affect the stability of DNMT1 or the ability of the mutant protein to restore genomic methylation levels when expressed in *Dnmt1*-null ES cells. Furthermore, both USP7 and PCNA are recruited to sites of DNA replication independently of the presence of DNMT1, and there is no evidence that DNMT1 is degraded in cycling cells after S phase.

**Conclusions:**

Multiple lines of evidence indicate that homeostasis of DNMT1 in somatic cells is controlled primarily at the level of transcription and that interaction of USP7 with the (GK) repeats of DNMT1 is unlikely to play a major role in the stabilization of DNMT1 protein.

## Background

**Fig. 1:**
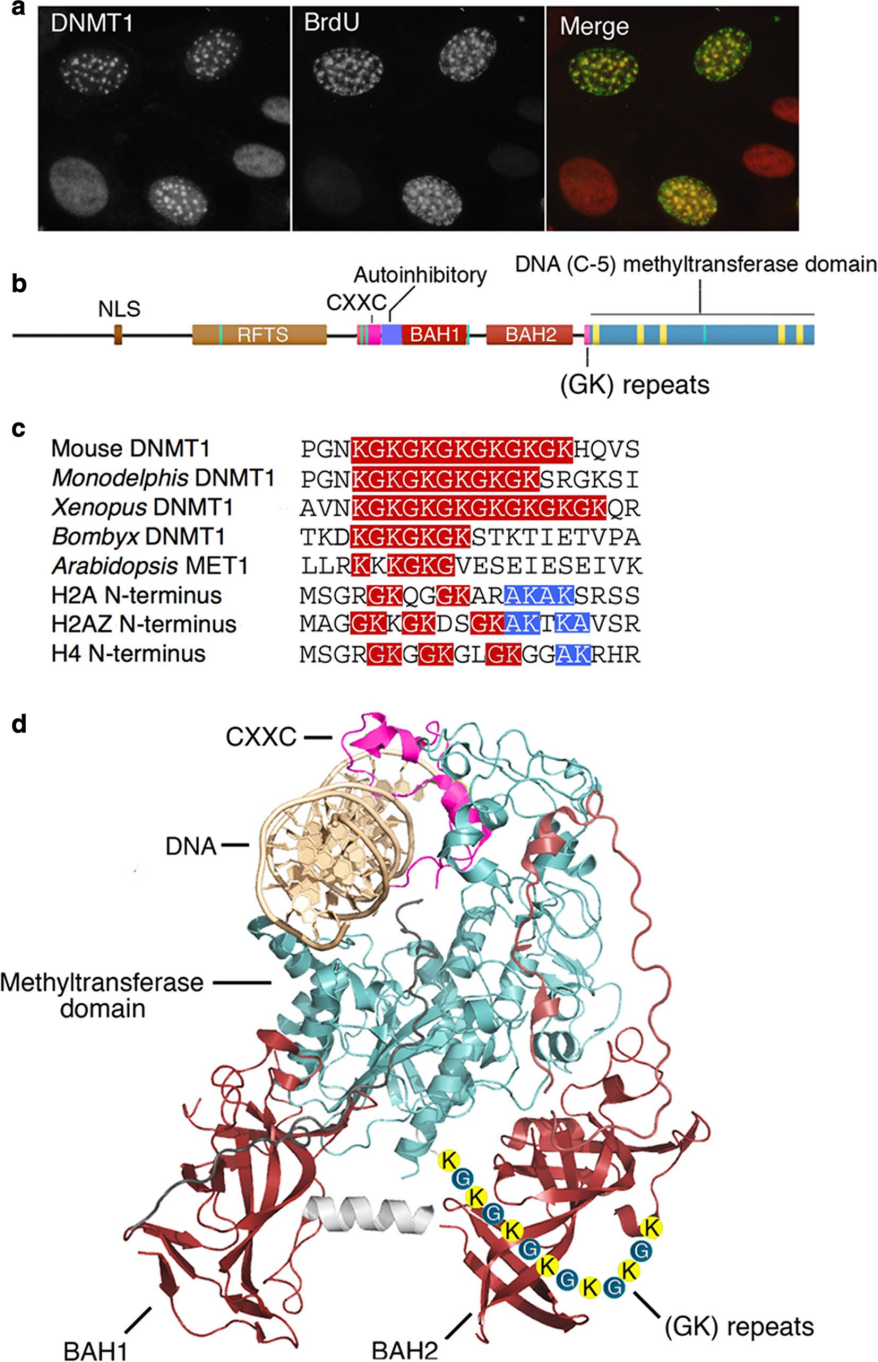
Cell-cycle independent expression of DNMT1 and the characteristics of the (GK) repeats in DNMT1. **a** Pulse labeling of cycling mouse 3T3 cell with BrdU followed by fixation and staining with antibodies to BrdU and DNMT1 shows that DNMT1 is present in non-S phase cells (identified by lack of BrdU staining) at levels comparable to S phase cells as previously reported [30]. **b** Domain organization of mammalian DNMT1. NLS: nuclear localization sequence. RFTS: replication focus targeting sequence. CXXC: Zinc-containing domain that binds to unmethylated CpG sites in double stranded DNA. Autoinhibitory: An acidic linker interposed between DNA and the active site of DNMT1 when the CXXC domain is bound to unmethylated DNA. BAH1 and 2: Bromo-adjacent homology domains that are of unknown function in DNMT1 but are involved in binding to specific histone modifications in other proteins. (GK): The run of alternating glycine and lysine amino acids at the junction between the N-terminal regulatory and C-terminal catalytic domains of DNMT1. Methyltransferase domain: Catalytic domain related in sequence and structure to eukaryotic and prokaryotic DNA (cytosine-5) methyltransferases. **c** Alignment of (GK) repeats of DNMT1 homologs from mammals, insects, and plants, and with the N-terminal tails of histones H2A, H2AZ, and H4. GK dipeptides are outlined in red; the related AK dipeptides are outlined in blue. **d** Structure of autoinhibited form of human DNMT1 in complex with unmethylated DNA to show spatial relationships of domains diagrammed in (**b**). The protein shown was truncated just N-terminal of the CXXC domain prior to crystallization. The (GK) repeats were unstructured in all the crystallographic studies of DNMT1; they are shown here roughly to scale in an arbitrary position

DNMT1 methylates hemimethylated CpG dinucleotides that appear after passage of the replication fork during S phase to ensure mitotic inheritance of genomic methylation patterns [1]. As shown in Fig. 1, DNMT1 bears a large N-terminal region that contains multiple functional domains that mediate nuclear import (the NLS domain), the suppression of de novo methylation (the CXXC and autoinhibitory domains) [2], two bromo-adjacent homology (BAH) domains of unknown function, and glycine-lysine (GK) repeats consisting of 13 alternating glycine and lysine residues. The (GK) repeats join the N-terminal regulatory domain to the C-terminal methyltransferase domain that is closely related in sequence and structure to DNA (cytosine-5) methyltransferases from both prokaryotes and eukaryotes [3]. The (GK) repeats are conserved among all eukaryotic DNMT1 homologs, all of which are lysine-rich and have at least one GK dipeptide between the regulatory and catalytic domains. GK dipeptides are also enriched in the N-terminal tails of histones H2A and H4 (Fig. 1c).

The structures of several mouse and human DNMT1 proteins have been determined [2, 4–6], and in all cases, the (GK) repeats are disordered in the crystal structure and not resolved, which implies that they do not form stable associations with other domains of DNMT1. The approximate positions of the (GK) repeats with respect to other domains of DNMT1 are shown in Fig. 1d. It has been reported that the (GK) repeats are involved in controlling proteasomal degradation of DNMT1 via reversible acetylation of lysines within the (GK) repeats [7–11]; this has been suggested to couple DNMT1 biosynthesis and degradation to the cell cycle [9]. Ubiquitin-specific protease 7 (USP7; also known as Herpes-associated ubiquitin-specific protease or HAUSP) [12–14] has been reported to bind to the unacetylated (GK) repeats of DNMT1 [10]; the acetylated form was reported to be incapable of binding to USP7 in vitro and has been proposed to undergo ubiquitylation at lysine 586 (RFTS domain) and lysine 997 (BAH2 domain) [15], and to be targeted for proteasomal degradation [7]. DNMT1 was also reported to be required for the recruitment of USP7 to sites of DNA replication in S phase nuclei [10]. These reports depended on the results of in vitro studies or transfection of tagged and mutated forms of DNMT1 into cells that contained endogenous DNMT1; the relative levels of endogenous and exogenous DNMT1 were not reported and only exogenous DNMT1 was observed. Furthermore, levels of DNMT1 do not change appreciably during the cell cycle [16], as shown in Fig. 1a, although DNMT1 is not expressed in G_0_ cells [17]. We report here that reduction in USP7 to undetectable levels in mouse and human cells did not cause a measurable reduction in content of DNMT1 or in DNA methylation, that substitution of the acetylated lysines within the (GK) repeats by glutamines does not affect the amount or activity of endogenous DNMT1, and that recruitment of USP7 to replication foci during S phase is independent of the presence of DNMT1. These and several other lines of evidence indicate that any USP7-DNMT1 interaction does not play a major role in the stabilization of DNMT1 and that levels of DNMT1 are regulated at the level of transcription.

## Methods

### Cell lines

Mouse ES cells as described in [18] were cultured on gelatin following standard techniques. Stable ES cell lines were generated by nucleofection of *Dnmt1*-null ES cells [18] with MT80 minigene and pGKPuro plasmid for Puromycin resistance [19]. MT80 minigene, carrying 12 kb of 5′ *Dnmt1* genomic sequence with endogenous promoter and 5.5 kb of *Dnmt1* cDNA [19, 20], was modified by the addition of an N-terminal Flag-HA tag after the translation start site. Point mutations were produced using QuikChange Site-Directed Mutagenesis kit (Agilent). Individual clones were selected with Puromycin for 10–14 days and picked into 96-well plates. Clones were genotyped using primers specific to the Flag-HA tag. Positive clones were propagated, and levels of DNMT1 expression were tested by Western blotting. Clones expressing DNMT1 at wild-type levels were used for further studies.

*Usp7* ^*cl/*+^ mice [13] were intercrossed to generate homozygous conditional mutant embryos. MEFs were derived and transfected with a construct that expressed SV40 large T antigen. MEFs were subsequently infected with Ad–GFP and Ad–Cre–GFP viruses from Vector Biolabs (Catalogue numbers 1761 and 1710). Cultures that showed near-complete infection with Ad-Cre-GFP virus as assessed by visualization of GFP expression were analyzed after five days.

To generate the inducible *Usp7* shRNA knockdown cells, H1299 cells were transfected with pTRIPZ encoding a Tet-inducible *Usp7* short hairpin RNA obtained from Thermo Open Biosystems (clone ID: V2THS_172409) and a Puromycin resistance cassette. 48 h later Puromycin (5 μg/ml) was added to the transfected cells for 14 days. To induce shRNA transcription, 5 μg/ml of doxycycline was added to the culture medium for 72 h prior to analysis.

### Immunoblotting

Whole cell extracts were prepared by lysis in RIPA buffer (150 mM NaCl, 1% NP-40, 0.5% Deoxycholic acid, 0.1% SDS, 50 mM Tris pH 7.5) and briefly sonicated to disrupt genomic DNA, then heated to 100° in SDS and loaded onto SDS-PAGE gels. Proteins were transferred to nitrocellulose membrane and blocked in 5% milk, 0.1% Tween 20, PBS for 1 h at room temperature. Blots were incubated at 4 °C overnight with primary antibodies in 10% FBS, 0.1% Tween 20. After incubation with DNMT1 antibody, blots were washed with PBST and PBS. Antibodies used: rabbit polyclonal to DNMT1 (Cell Signaling Technology, 5032 (D63A6) 1:2500 dilution), rabbit polyclonal to USP7 (Bethyl Laboratories, IHC00018; 1:5000 dilution), rabbit polyclonal to HA tag (Abcam, ab9110, 1:5000), rabbit polyclonal to UHRF1 (Bethyl Laboratories, A301-470A, 1:1000), mouse monoclonal to alpha-tubulin (Abcam, ab7291, 1:10,000).

Three independent biological replicates were performed. DNMT1 and tubulin levels were quantified using ImageJ. Statistical analysis was performed using the two-tailed *t* test.

### Methylation analysis

Genomic DNA was extracted by digestion with proteinase K and RNase A followed by phenol/chloroform extraction and isopropanol precipitation. Genomic DNA was digested for two rounds with methylation-sensitive enzyme HpaII, its isoschizomer MspI as a control, or McrBC (all from NEB). DNA was quantified and ran on 0.8% agarose gel, which was stained with ethidium bromide.

Southern blot analysis was performed with IAP probes generated by PCR. Primers used for probe amplification: probe IAP F (GGTAAACAAATAATCTGCGC); probe IAP R (CTGGTAATGGGCTGCTTCTTCC). DNA in agarose gels was transferred to a Nytran SPC membrane (GE Healthcare) overnight in 10 × SSPE buffer. After crosslinking, membrane was prehybridized with 6X SSC, 5X Denhardts, 1% SDS, 10% Dextran Sulfate for 1 h at 45° and incubated overnight with IAP probe at 45°. Membranes were washed once with 2X SSC, 0.5% SDS; 2X with 1X SSC, 0.5% SDS, and 1X with 0.2X SSC, 0.5% SDS.

Global levels of DNA methylation were quantified using LUminometric Methylation Assay (LUMA) as described previously [21]. 400 ng of genomic DNA was digested with MspI/EcoRI and HpaII/EcoRI in parallel. The overhangs from the enzymatic digestion were quantified by Pyrosequencing (PyroMark Q24, Qiagen) with the dispensation order: GTCACAGTGT. Global DNA methylation levels were calculated from the peak heights at positions 3,4,7,8 using the following formula:

$${\text{Global}}\;{\text{methylation}}\; (\% )= \left[ {1 - \left( {{\text{HpaII}}\;\varSigma G/\varSigma T} \right)/\left( {{\text{MspI }}\varSigma G/\varSigma T} \right)} \right] \times 100$$

Statistical analysis was performed on biological replicates using the two-tailed *t* test.

### Immunofluorescence

ES cells were cultured on glass slides. For the PCNA immunostaining, cells were treated with 0.5% Triton X-100 in CSK buffer (100 mM NaCl, 300 mM sucrose, 10 mM PIPES [piperazine-*N*,*N*-bis(2-ethanesulfonic acid)], pH 6.8, 3 mM MgCl2, 1 mM EGTA) for 30 s at 4 °C, and then treated with methanol for 20 min at − 20 °C [22].

Cells were permeabilized/blocked in Block solution (5% Donkey serum, 0.3% Triton, 1X PBS) and then incubated overnight with primary antibody diluted in Block solution at 4 °C. The following primary antibodies were used: mouse monoclonal to PCNA (Abcam, ab29, 1:500 dilution), rabbit polyclonal to USP7 (Bethyl Laboratories, IHC00018; 1:100 dilution).

Cells were washed ten times with PBS and incubated for 1 h at room temperature with the following secondary antibodies diluted in Block buffer: Cy3-conjugated donkey anti-mouse (Jackson ImmunoResearch; 1:200 dilution) and IgM Alexa 488–conjugated IgG donkey anti-rabbit (Jackson ImmunoResearch; 1:500 dilution). Slides were subsequently washed with PBS, counterstained with Hoechst 33,342 (Invitrogen) and mounted with Vectashield mounting medium (Vector Labs).

### Statistical analysis

Statistical data were calculated for groups with normal distributions and similar variances. Variation within each group of data is reported as standard deviation. ‘*n*’ represents the number of biological replicates. *p* values were calculated using the two-tailed *t* test. The R program was used to ensure that *n* = 2 was sufficient to establish a power of 0.8.

## Results

### Ablation of USP7 does not affect stability or function of DNMT1

**Fig. 2:**
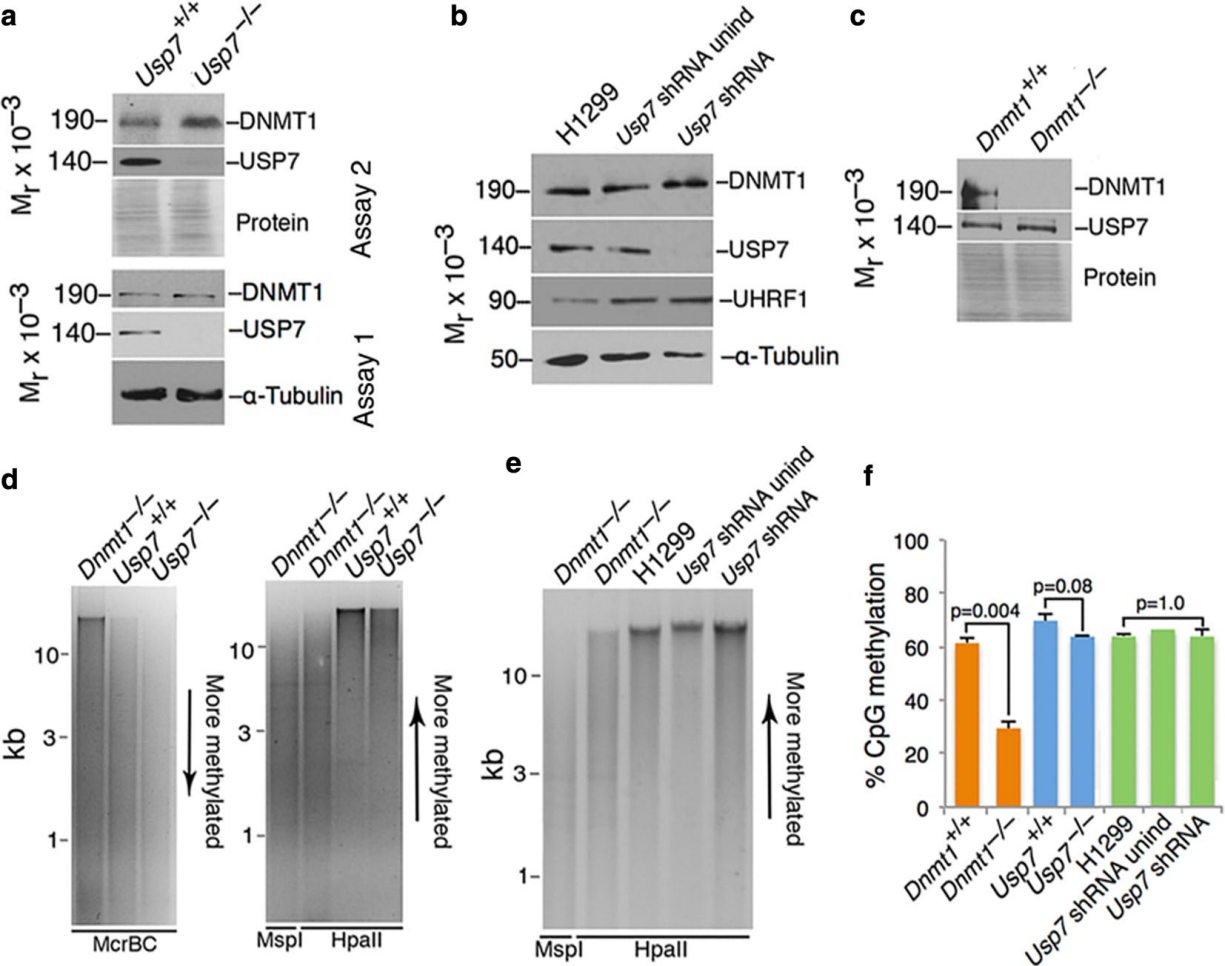
Ablation of USP7 does not affect steady-state level of DNMT1 or global DNA methylation. **a** MEFs that lack USP7 after Cre-mediated excision of a Floxed allele of *Usp7* show normal levels of DNMT1 (lane 2) but no detectable USP7 protein. Biological replicates are shown as Experiments 1 and 2. **b** Normal expression of DNMT1 and UHRF1 in human H1299 lung carcinoma cells containing an inducible shRNA against USP7 mRNA. Ablation of USP7 has no detectable effect on expression of other proteins. **c** Normal steady-state expression of USP7 in the absence of DNMT1 in ES cells. **d** Normal DNA methylation in MEFs that lack USP7. McrBC cleaves methylated DNA; HpaII cleaves unmethylated DNA at CCGG sites; MspI cleaves CCGG sites whether methylated or unmethylated. DNA from wild-type and *Usp7*^−*/*−^ MEFs show similar patterns of resistance to both McrBC and HpaII. **e** Removal of USP7 by an inducible shRNA against USP7 from H1299 human lung carcinoma cells does not affect DNA methylation levels as assessed by resistance of DNA to HpaII. **f** LUMA analysis shows that DNA methylation is not measurably affected by removal of USP7. *n* = 2 (biological replicates). Error bars show standard deviations, center value is mean, and *p* values were calculated using the two-tailed *t* test

Mouse embryonic fibroblasts (MEFs) homozygous for Floxed alleles of *Usp7* were transfected with an expression construct that encoded SV40 large T antigen, then infected with a recombinant Adenovirus that drives expression of GFP and Cre recombinase. Cultures that showed near-complete infection as measured by GFP expression were cultured for 3–5 days and evaluated for expression of USP7 and DNMT1 by immunoblot. As shown in Fig. 2a, levels of DNMT1 were not affected when USP7 was reduced to undetectable levels. Three independent biological replicates were performed, and DNMT1 and tubulin levels were quantified using ImageJ. Statistical analysis was performed using the two-tailed *t* test, and the p value was > 0.7. USP7 was present at normal levels in embryonic stem (ES) cells null for *Dnmt1* (Fig. 2c). Global methylation levels were assessed by digesting genomic DNA with the methylation-sensitive restriction endonuclease HpaII, its isoschizomer MspI as a control, and McrBC, which digests methylated DNA. Methylation levels were quantified by LUminometric Methylation Assay (LUMA) that uses methylation-sensitive enzymes HpaII and MspI followed by Pyrosequencing which quantitates the number of HpaII cleavage events [21]. As shown in Fig. 2d, global levels of DNA methylation were not notably affected by the removal of USP7, which is consistent with the lack of effect on DNMT1 levels upon removal of USP7. The human lung carcinoma cell line H1299 was transfected with an expression cassette that drives expression of an inducible USP7 shRNA and a stable cell line was derived. The shRNA produced is complementary to nucleotides 2766–2748 of transcript variant 1 and caused strong repression of all *Usp7* transcript variants. This inducible system allows simultaneous induction of shRNA within the entire cell population. As shown in Fig. 2b, shRNA-mediated reduction in USP7 to undetectable levels did not affect expression of DNMT1 or UHRF1. Figure 2e shows that global DNA methylation was not measurably affected by the loss of USP7 expression. Figure 2f shows quantification of genomic methylation levels by LUMA. MEF cells lacking USP7 showed similar methylation to wild-type MEFs (*p* = 0.08), and H1299 cells with severe reduction in USP7 levels exhibit no difference in methylation as compared to control H1299 cells (*p* = 1.0). These genetic data indicate that removal of USP7 does not lead to a significant reduction in DNMT1 or in global DNA methylation.

### Mutation of GK repeats results in normal stability and activity of DNMT1

**Fig. 3:**
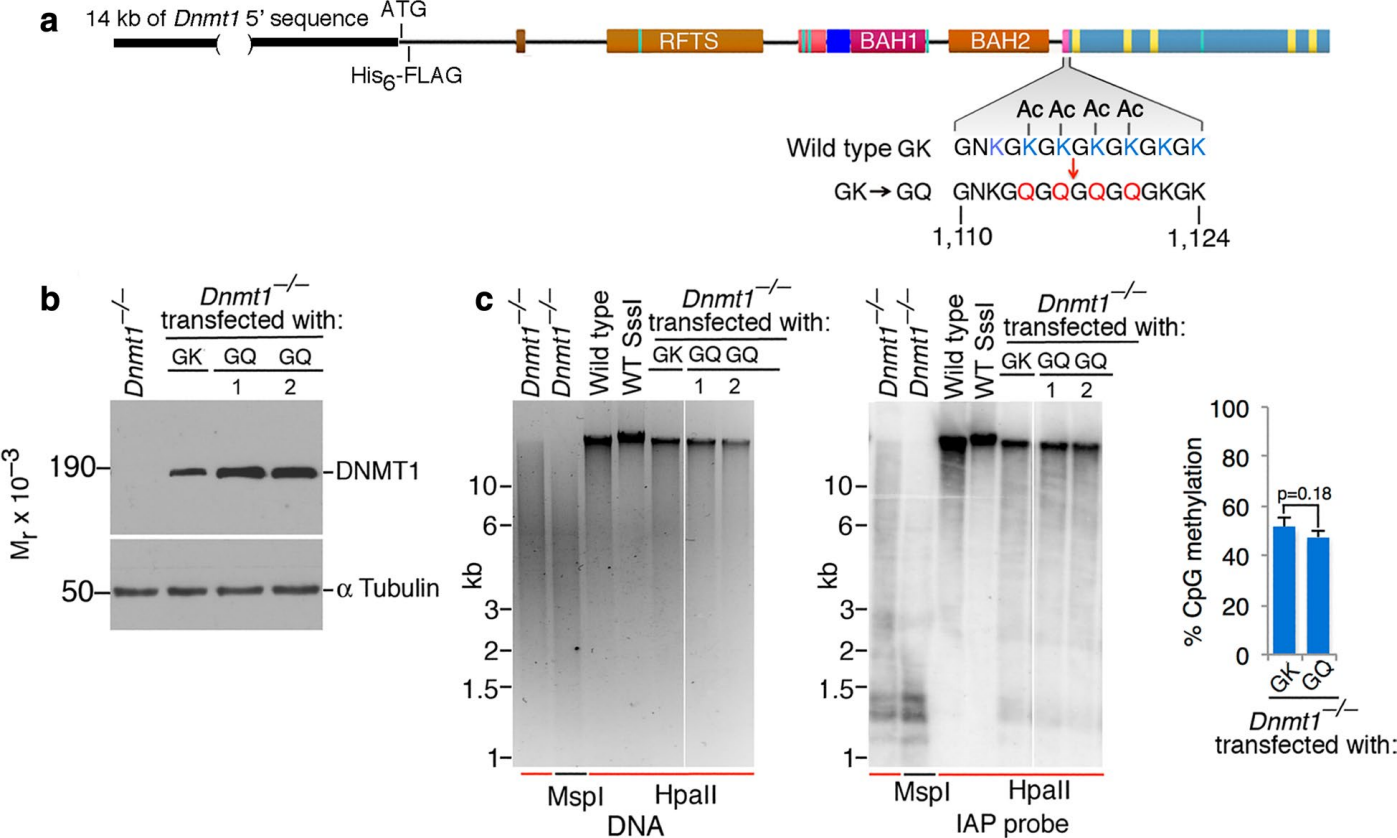
Substitution of lysines by glutamines within (GK) repeats does not affect DNMT1 stability or function. **a** Positions of GK→GQ substitutions within the (GK) repeats of DNMT1. The substituted lysines correspond to those reported to be acetylated in vivo and to block the interaction of DNMT1 and USP7 in vitro. **b** Normal stability of GK→GQ DNMT1 in stably transfected *Dnmt1*-null ES cells. Two independent transfected clones are shown. **c** Normal methylation of genomic DNA as measured by resistance to HpaII in stably transfected ES cells at left; at center, equal methylation of IAP retrotransposon DNA by wild-type and GK→GQ DNMT1. “WT SssI” indicates DNA that had been methylated at all CpG sites by treatment with M.SssI. At right is LUMA analysis of DNA methylation in (GK) versus (GQ) DNMT1 expression cells. *n* = 3 (biological replicates). Error bars indicate standard deviations. The center value is mean, and *p* values were calculated using the two-tailed *t* test

To further investigate the role of the reported interaction of USP7 with the (GK) repeats of DNMT1 [10], we substituted the lysine residues within the (GK) repeats that have been reported to undergo acetylation [22, 23] with glutamines (Fig. 3a), which cannot be acetylated and have been proposed to mimic acetylated lysines and to block the in vitro interaction of USP7 and DNMT1 [10]. Expression constructs that directed production of wild type (GK) or (GK)**→**(GQ) DNMT1 were transfected into *Dnmt1*-null ES cells and stable clones established as described [19]. Expression was driven by 12 kb of DNA 5′ of the *Dnmt1* gene, which contains the *Dnmt1* promoter and other regulatory sequences and has been shown to drive expression of DNMT1 to wild-type levels [19, 20]. As shown in Fig. 3b, the (GK) and (GQ) proteins were expressed at similar levels, even though the (GQ) substitution has been reported to be unable to bind to USP7 and according to previous reports would have been expected to be degraded [10]. Figure 3c also shows that the GK and GQ proteins were equally effective in rescuing global DNA methylation after expression in *Dnmt1*-null ES cells and that rescue of methylation of the IAP retrotransposon is equivalent when the (GK) and (GQ) variants of DNMT1 are expressed. Methylation levels by LUMA assay showing no significant difference between (GK) and (GQ) rescued ES cells (*p* = 0.18). The data of Figs. 2 and 3 indicate that the steady-state levels of DNMT1 are independent of USP7, that removal of USP7 does not affect global DNA methylation, and that substitution of the lysines in the (GK) repeats of DNMT1 that have been reported to be required for the DNMT1-USP7 interaction and stabilization of DNMT1 [10] do not affect the expression or biological function of DNMT1.

### USP7 localizes to replication foci independently of DNMT1

**Fig. 4:**
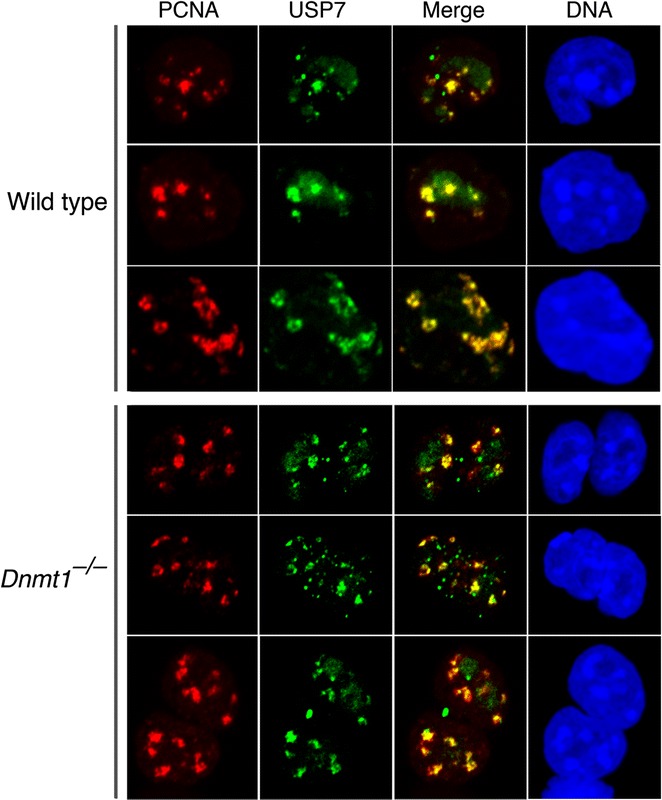
USP7 is recruited to replication foci in the absence of DNMT1. Replication foci where identified by staining for the replication factor PCNA. Colocalization of USP7 and PCNA is clearly apparent in both wild type (top three rows) and *Dnmt1*-null (bottom three rows) ES cell nuclei

It was reported that the recruitment of USP7 to replication foci is dependent on the (GK) repeats of DNMT1 [10]. We tested for recruitment of USP7 to replication foci in ES cells that were wild type or null for *Dnmt1*. As shown in Fig. 4, USP7 colocalized with the replication focus marker PCNA independently of DNMT1. These data show that USP7 is not recruited to replication foci by DNMT1 and are consistent with a recent report in which USP7 is required to maintain an enrichment in SUMOylation and depletion of ubiquitylation at sites of DNA replication that is essential for DNA replication [15] and for the removal of minichromosome maintenance complex binding protein (MCM-BP) from chromatin after DNA replication [24], which occurs after completion of maintenance DNA methylation. DNMT1 is not required for DNA replication, as shown by normal DNA replication in *Dnmt1*-null ES cells [19].

## Discussion

Several lines of evidence indicate that USP7 does not control cell cycle-dependent levels of DNMT1 in vivo as had been claimed. First, DNMT1 is not degraded after S phase in cycling cells; in Xenopus extracts DNMT1 levels and chromatin loading are unperturbed upon the inhibition of proteasome activity [25]. Second, DNMT1 levels are independent of the presence of USP7, as shown in Fig. 2. Third, substitution of the GK repeats with GQ repeats, which prevents the acetylation of DNMT1, does not affect steady-state levels of DNMT1 (Fig. 3). Fourth, USP7 localizes to replication foci independently of DNMT1 (Fig. 4). Fifth, modification of the endogenous *Dnmt1* locus so as to delete the first 118 amino acids of DNMT1 caused the accumulation and persistence of truncated but fully active DNMT1 to ~ 5 times normal levels [26]. This is the only genetically defined region of DNMT1 that affects protein stability. However, this shortened and stabilized form of DNMT1 is produced only in growing oocytes and has not been detected in somatic cells [27].

The (GK) repeats are both highly basic and unstructured; they are therefore capable of adopting many conformations and would be expected to bind nonspecifically in vitro to many proteins that contain acidic pockets, as is the case for USP7. Furthermore, prior studies expressed tagged recombinant DNMT1 in wild-type cells where the relative amounts of tagged recombinant DNMT1 to endogenous wild-type DNMT1 were not reported [10].

Despite the numerous reports of post-translational regulation of DNMT1 expression in an acetylation-dependent manner [7–11], strong evidence indicates that DNMT1 levels are normally controlled at the transcriptional rather than the post-translational level. Cells heterozygous for loss-of-function mutations at *Dnmt1* express one-half the amount of DNMT1 protein when compared to wild-type cells [28], and mice that contain additional copies of the *Dnmt1* gene overexpress DNMT1 protein [29]. DNMT1 is overexpressed in Friend Murine Erythroleukemia cells as a result of spontaneous amplification of the *Dnmt1* gene in this cell type [3]. DNMT1 is present in nuclei at constant levels throughout the cell cycle and is recruited to replication foci during S phase [30]. DNMT1 is down-regulated in G_0_ cells, but this is likely to occur at the transcriptional level [17]. Treatment of cells with drugs that induce entry into G_0_ phase will cause a reduction in DNMT1 levels due to cell cycle effects rather than a direct effect on DNMT1 stability [17]. There is no direct evidence of a post-translational mechanism that compensates for reduced or increased DNMT1 transcript levels, and changes in *Dnmt1* gene dosage result in proportionate increases or decreases in DNMT1 protein level.

Many studies have tried to identify regulators of DNMT1 using immunoprecipitation assays; the only confirmed regulator of DNMT1, the E3 ubiquitin ligase UHRF1, was identified in a genetic screen, not an interaction screen. Null alleles of *Uhrf1* phenocopy null alleles of *Dnmt1* in mice [31]. Recent studies suggest that UHRF1 acts through binding and ubiquitylation of histones and other proteins at the replication foci [32, 33] rather than by effects on DNMT1 expression.

## Conclusions

These findings indicate that the steady-state level of DNMT1 protein in cycling cell populations is controlled at the level of transcription and that the interaction of DNMT1 and USP7 is unlikely to play a major role in DNMT1 homeostasis.
